# Cold Physical Plasma Modulates p53 and Mitogen-Activated Protein Kinase Signaling in Keratinocytes

**DOI:** 10.1155/2019/7017363

**Published:** 2019-01-13

**Authors:** Anke Schmidt, Sander Bekeschus, Katja Jarick, Sybille Hasse, Thomas von Woedtke, Kristian Wende

**Affiliations:** ^1^Leibniz-Institute for Plasma Science and Technology e. V., Felix-Hausdorff-Str. 2, 17489 Greifswald, Germany; ^2^ZIK Plasmatis at Leibniz-Institute for Plasma Science and Technology e. V, Felix-Hausdorff-Str. 2, 17489 Greifswald, Germany; ^3^Department of Hygiene and Environmental Medicine, University Medicine Greifswald, 17475 Greifswald, Germany

## Abstract

Small reactive oxygen and nitrogen species (ROS/RNS) driven signaling plays a significant role in wound healing processes by controlling cell functionality and wound phase transitions. The application of cold atmospheric pressure plasma (CAP), a partially ionized gas expelling a variety of ROS and RNS, was shown to be effective in chronic wound management and contrastingly also in malignant diseases. The underlying molecular mechanisms are not well understood but redox signaling events are involved. As a central player, the cellular tumor antigen p53 governs regulatory networks controlling proliferation, death, or metabolism, all of which are grossly modulated by anti- and prooxidant signals. Using a human skin cell model, a transient phosphorylation and nuclear translocation of p53, preceded by the phosphorylation of upstream serine- (ATM) and serine/threonine-protein kinase (ATR), was detected after CAP treatment. Results indicate that ATM acts as a direct redox sensor without relevant contribution of phosphorylation of the histone A2X, a marker of DNA damage. Downstream events are the activation of checkpoint kinases Chk1/2 and several mitogen-activated (MAP) kinases. Subsequently, the expression of MAP kinase signaling effectors (e.g., heat shock protein Hsp27), epithelium derived growth factors, and cytokines (Interleukins 6 + 8) was increased. A number of p53 downstream effectors pointed at a decrease of cell growth due to DNA repair processes. In summary, CAP treatment led to an activation of cell repair and defense mechanisms including a modulation of paracrine inflammatory signals emphasizing the role of prooxidant species in CAP-related cell signaling.

## 1. Introduction

Cold physical plasma (CAP) is an emerging biomedical technique and found to interfere with processes controlled by redox signaling *in vivo*, such as wound healing, immune modulation, and cancer [1–6]. Different source geometries and discharge types (barrier discharges and plasma jets) have been developed with some being accredited medical products [7–10]. In principle, energy is introduced into a noble gas or a molecular gas resulting in the ionization of a fraction of it. CAP expels various forms of energy: light (UV, visible, and IR), electromagnetic fields, and small chemical entities like electrons, ions, and molecules. To the current knowledge, the short- and long-lived reactive oxygen and nitrogen species (e.g., NO•, •O_2_^−^, N_x_O_y_, HO•, ONOO^−^, and H_2_O_2_) are the major contributors to the described effects both *in vitro* and *in vivo*. Their occurrence can be controlled by plasma source design and discharge parameter engineering, especially the composition of the working gas. In vivo, stimulatory effects in chronic or acute wound healing (animal models) or in humans (clinical trials) were reported [5, 11–14]. The biochemical background of these observations is so far not fully investigated. In vitro, an impact on cell viability and cell cycle progression is frequently observed, along with changes in cell metabolism, redox signaling, and protein secretion [15–20]. Increasing knowledge is gathered regarding cellular redox signaling pathways [21, 22]. The cellular tumor antigen p53 is basically a transcription factor with a major role in DNA damage sensing and control, hypoxia, or nutrient fluctuation [23]. Further roles have been reported, e.g., interaction with apoptotic effectors in the cytosol [24, 25]. A wide range of posttranslational modifications adjusts p53's transcriptional and transcription-independent functions. Accordingly, p53 regulation is fundamentally involved in processes requiring cell repair, cell proliferation, or cell migration, such as acute and chronic wounds [26–28].

A complex, not fully understood relationship evolved around p53 and redox signal transduction checking free reactive oxygen or nitrogen species (ROS/RNS) levels and controlling cell fate [29]. The activation of p53 sparks both pro- and antioxidant downstream effects. Prooxidant measures promote autophagy and apoptosis, while antioxidant effects comprise increased protein expression involved in NADPH and glutathione metabolism, mitochondrial membrane stabilization, and modulation of nuclear factor- (erythroid-derived 2-) like 2 (NFE2L2 or NRF2) related signaling [30–32]. This major antioxidant pathway is protective against oxidative damages and is activated by natural and xenobiotic triggers, e.g., photodynamic therapy, small molecules like sulforaphane, or cold atmospheric plasma (CAP) as source for ROS/RNS [16, 33, 34].

Published data on CAP effects in cells or tissues suggest a role for p53, its downstream targets, and connected pathways such as the mitogen-activated protein (MAP) kinases in governing the cellular response towards CAP-derived ROS/RNS [35]. Potentially, via oxidative signals, an activation of major MAP kinases [36] precedes a kinase-driven posttranslational modification of p53 activity [37–39] and establishes a cross-talk between the MAP kinase and the p53 signaling pathways [40–42], even influencing cell migration [43]. To test this hypothesis, a well-described human epithelial model cell line (HaCaT) was used to analyze p53 phosphorylation, activation of up- and downstream targets of the p53, and the expression of related genes or proteins in response to CAP.

A strong activation of the MAPK – p53 axis was found after CAP, emphasizing that plasma-derived ROS/RNS have a significant impact on cell fate and performance. The involvement of p53 phosphorylation indicates a substantial impact on cellular processes necessary to adapt to CAP treatment. An activation of cell protective processes accompanied by an increased expression of growth factors and cytokines relevant in wound underlines the use of CAP in wound management and other redox-signaling related conditions.

## 2. Materials and Methods

### 2.1. Cell Culture Cells and Cold Plasma Treatment

HaCaT keratinocytes were cultivated in RPMI 1640 cell culture medium containing 8% fetal bovine serum (Sigma-Aldrich, Germany), 2 mM glutamine, 0.1 mg/ml streptomycin, and 100 U/ml penicillin (PAN Biotech, Germany) at 37°C, 95% relative humidity, and 5% CO_2_ [16]. Twenty-four hours prior to experiment, 1 × 10^6^ cells were seeded in 60 mm dishes (Sarstedt, Germany). As cold physical plasma source, the kINPen 09 (neoplas tools, Germany) was utilized. This plasma jet consists of a central pin-type electrode that ignited a plasma by applying a voltage of 2–6 kV at a frequency of around 1 MHz. Argon (Air Liquide, France) was used as feed gas (3 standard liters per minute). For all experiments, an indirect treatment regimen was chosen to assure homogeneity of the treatment and it was achieved by exposing 5 ml of RPMI w/ all supplements to the plasma effluent at a distance of 9 mm using an automated xyz-table. The treated liquid was transferred immediately to the prepared cells.

### 2.2. Cellular Viability, ROS Levels, and Apoptosis

Changes of intracellular redox levels were determined using CM-H_2_DCF-DA (Life Technologies, CA, USA). Cells were stained with 1 *μ*M of dye for 20 min, prior to plasma-treated medium was added, and evaluated 5 min thereafter by fluorescence microscopy. Cell viability was assessed using the CellTox™ Green Cytotoxicity Assay (Promega, Germany). Briefly, 15,000 cells were seeded in 96 well plates 24 h prior to experiment. 24 h after indirect treatment, the dye was added. After 15 min, the fluorescence intensity was measured at *λ*_ex_ 490 nm and *λ*_em_ 525 nm using a microplate reader (Infinite 200, Tecan, Switzerland). To determine late apoptosis, a *Gallios* flow cytometer and *Kaluza* software (Beckman Coulter, CA, USA) were applied 18 hours after indirect treatment using Green Caspase-3 Kit (Promokine, Germany) according to the manufacturer's protocol.

### 2.3. Immunofluorescence Microscopy

HaCaT cells were grown on glass coverslips for 24 h, and plasma treated as indicated. After appropriate time points, cells were fixated with 4% (*w*/*v*) paraformaldehyde for 20 min, permeabilized with PBS/0.1% Triton X-100 for 20 min, and treated with 3% bovine serum albumin (BSA) in PBS. Slides were washed twice with 1% BSA in PBS and incubated at 4°C overnight with p53 antibody (NEB, Germany, 1 : 1.600). Afterwards, cells were washed twice with PBS and incubated with secondary antibody Alexa Fluor 488-conjugated goat anti-rabbit (Life Technology, Germany, 1 : 700) for 1 h. Coverslips were washed again with PBS and mounted using VECTASHIELD® with DAPI (Vector Labs, CA, USA). Samples were observed using an Axio Observer Z1 (Zeiss, Germany).

### 2.4. Gene Expression Analysis by Quantitative Real-Time PCR

RNA was isolated using RNA Mini Kit (Bio&SELL, Germany), and total mRNA was reversely transcribed using Transcriptor First Strand Synthesis Kit (Roche, Germany). Primer specificity was confirmed by separating PCR amplification products in an agarose gel. Quantitative real-time PCR was performed using the Fast Sybr Kit (Kapa Biosystems, MA, U.S.A.) and a LightCycler 480 (Roche, Germany). Gene specific primers for BAX, BBC3, GADD45, and CDKN1A were used [44–47] at a concentration of 200 nM (Suppl.  Table 1). The samples were preincubated at 95°C for 3 min, followed by 40 amplification cycles of 10 s denaturing at 95°C, 30 s annealing at 55°C, and amplification for 1 s at 72°C. Finally, a melting curve was performed with five acquisitions/°C from 65°C to 97°C. All samples were performed in triplicates. To calculate relative gene expression, the data of the threshold cycles was analyzed using the ΔΔC_T_ method.

### 2.5. Western Blot and ELISA

Cells were plasma-treated, rinsed with ice-cold PBS, and then lysed in ice-cold RIPA lysis buffer containing protease and phosphatase inhibitors (cOmplete/PhosSTOP; Roche, Germany) and 2 mM phenylmethanesulfonylfluoride (PMSF; Carl Roth, Germany). The protein concentrations were equalized and samples were heated to 95°C for 5 min in Laemmli buffer (0.25 mM Tris, 2% SDS, 10% glycerol, 2% *β*-mercaptoethanol, 0.001% bromophenol blue). Proteins were separated on a 10% SDS-PAGE Gel (Anamed GmbH, Germany) and blotted onto a Roti®PVDF membrane (Carl Roth, Germany). After blocking in TBS-T (0.05% nonfat milk powder in TRIS-buffered saline pH 7.6/0.05% Tween 20,TBS-T), blots were incubated with Erk1/2 (#9102), Mek1/2 (#9126), Sapk/Jnk (#9258), p38 (#9212), p53 (#2527) as well as phospho-specific antibodies for p-ATM (S1981, #5883), p-ATR (S428, #2853), p-Chk1 (S296, #2349), p-Chk2 (T68, #2661), p-Erk1/2 (T202/Y204, #4370), p-p38 (T180/Y182, #9216), p-Mek1/2 (S217/S221, #9154), p-Sapk/Jnk (T183/Y185, #4668), p-HSP27 (S78,# 2405), p-p53 (S15, # 9286), and p-p53 (S37, #2989), all 1 : 1000 in TBS-T at 4°C overnight (Cell Signaling Technologies, Germany). Then, procedure was preceded by 1 h incubation with secondary antibody (Jackson Europe, UK) 1 : 10,000 in TBS-T and followed by incubation with ECL reagent. Chemiluminescence was detected by ImageQuant LAS 4000 and analyzed by ImageQuantTL (GE Healthcare, UK). Phosphorylated protein levels of p53-dependent kinases were normalized to *β*-actin (housekeeping). Analyses of secreted proteins were performed using the enzyme-linked immunosorbent assay (ELISA). Human IL-6, IL-8, and GM-CSF were detected using ELISA Max™ kits (BioLegend, UK) and human VEGF-A using ELISA (Thermo Scientific, Germany). Procedures were performed according to the manufacturers protocols.

### 2.6. Statistical Analysis

At least three independent experiments were performed in all assays. Bar graphs represent arithmetic mean + standard deviation (S.D.). Statistical comparison between experimental groups was done using one-way analysis of variances followed by *Dunnett* posttesting comparing treated samples to untreated control samples. When investigations were carried out at different time points, statistical analysis was done for each time point independently. A *p* value of ≤0.05 was considered statistically significant.

## 3. Results

### 3.1. Intracellular ROS, Cell Viability, and Apoptosis

**Figure 1:**
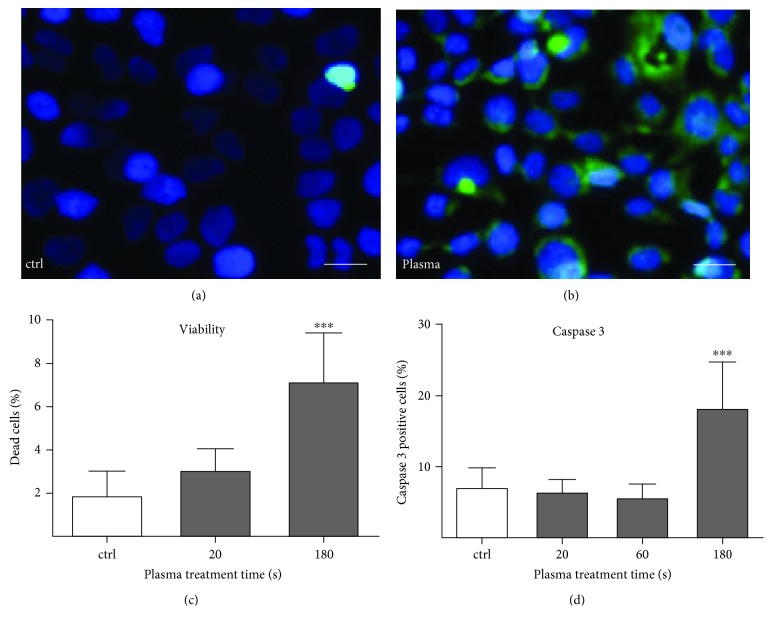
Cold plasma oxidized keratinocytes and altered cell viability. The intracellular ROS level was detected by CM-H_2_DCFDA fluorescence staining for control (a) and indirectly plasma-treated HaCaT keratinocytes (using kINPen 09 plasma jet) (b). For assessment of cell viability, the CellTox™ Green Dye was used and showed a 1.5 to 3.5-fold increase of death cells after 20 s or 180 s of plasma treatment, respectively (c). To quantitate apoptosis, plasma-treated cells were stained with active caspase 3-detecting reagents and examined by flow cytometry. A significant 2.1-fold of caspase 3-positive cells was detected after 180 s of plasma treatment (d). Data are presented as mean + S.E. of four independent experiments; statistical comparison was done using one-way ANOVA (^∗∗∗^ *p* < 0.001). Scale bar 50 *μ*m.

Microscopic evaluation of the HaCaT cells after treatment showed an increased fluorescence signal of the redox-sensitive dye CM-H_2_DCF (Figures 1(a) and 1(b)`). This increased ROS prevalence could also be detected in a treatment time-dependent manner by flow cytometry using the same dye (data not shown). After 24 h, a significant 3.5-fold increase in dead cell numbers was detected for high-treatment intensity 180 s (Figure 1(c)). In parallel, the late apoptosis marker caspase 3 activity increased significantly to 18% (Figure 1(d), basal level ≈ 6–8%). Early apoptotic signs like phosphatidylserine externalization remain scarce (data not shown).

### 3.2. Plasma-Induced Accumulation and Nuclear Translocation of the Tumor Suppressor p53

**Figure 2:**
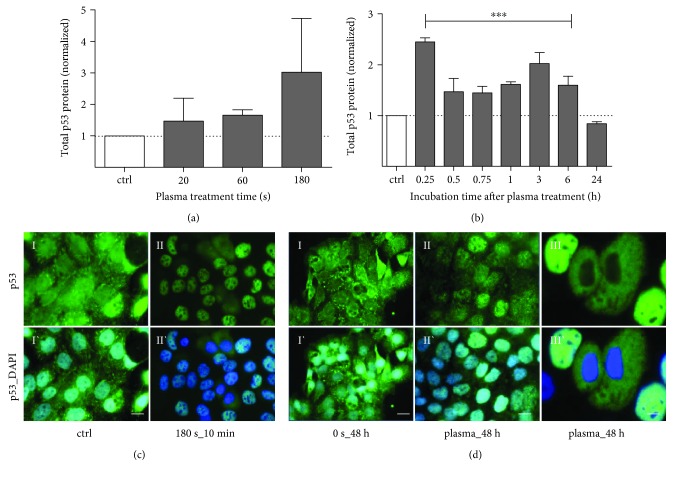
Cold plasma transiently enhanced total p53 protein expression and induced nuclear translocation. Total expression of p53 showed a treatment time-depending increase (a, after 3 h), in particular, 3 h after plasma exposure (b, 180 s). Immune fluorescent microscopy of HaCaT cells revealed a strong translocation of p53 (green) from cytoplasm into the nucleus in dependence of treatment and incubation time (CII) in contrast to control (CI). After 30 min, p53 was exclusively detected in nuclei. Forty-eight hours after plasma exposure, p53 was redistributed in the cytoplasm of HaCaT cells. Data are presented as mean + S.D. of two analyses (a, b) or as one representative (c, d). Statistical analysis was done using one-way ANOVA with *Dunnett* corrections for multiple comparisons to untreated, normalized control (^∗∗∗^ *p* < 0.001). Scale bar 50 *μ*m (CII, DI-II) and 20 *μ*m (CI, DIII).

Three hours after plasma, a treatment time-depending increase of total p53 protein expression was observed (Figure 2(a)). On a timeline, p53 protein expression levels fluctuated with peaks 15 min (2.3-fold) and 3 h (1.9-fold) after treatment and returned to the baseline level within 24 h (Figure 2(b), 180 s of treatment). Immunofluorescence staining with an anti-p53 antibody showed the subcellular localization of endogenous total p53 after plasma exposure (Figure 2(c)). While control cells showed a predominant localization of p53 in the cytosol (Figure 2(c), I), an immediate and rapid cytoplasmic-nuclear trafficking was observed already 10 min after treatment (Figure 2(c), II). The nuclear localization of p53 was observed up to 24 h after treatment, changing to a predominantly cytoplasmic distribution about 48 h after treatment (Figure 2(d)).

### 3.3. Plasma Treatment Contributes to p53 Phosphorylation on Serine 15 and 37

**Figure 3:**
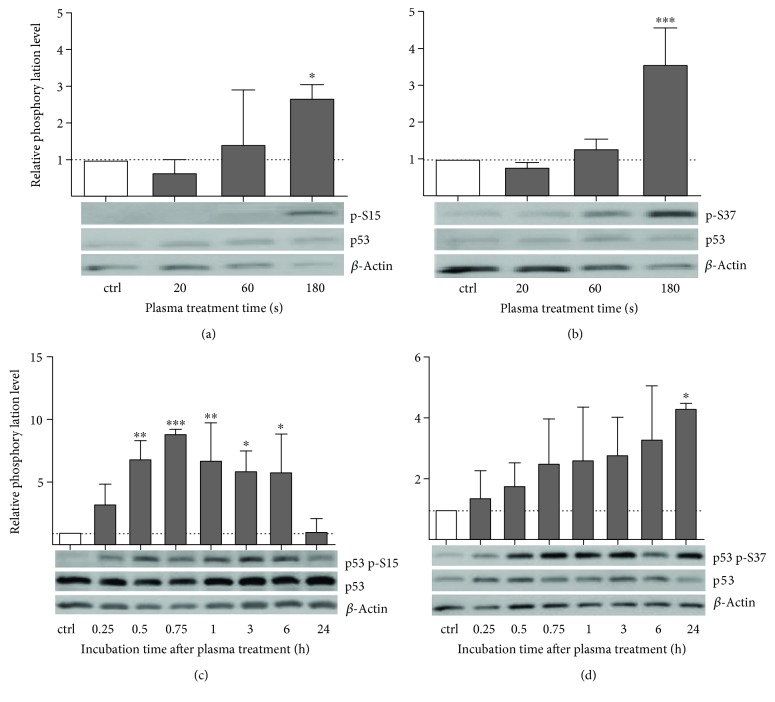
Cold plasma alters phosphorylation level of p53 in a treatment and incubation time-dependent manner. The upper graphs showed the treatment time-dependent activation of p53. Displayed are relative p53 phosphorylation levels of residues Ser15 (a) and Ser37 (b) normalized to total p53 and *β*-actin expression. Bottom graphs displayed the time courses of relative phosphorylation after longest plasma treatment of relative p53-Ser15 (c) and Ser37 phosphorylation (d). Untreated samples were included as negative control (ctrl). Data are presented as mean + S.D. of two independent experiments. The *x*-axis represents treatment time (a, b) or incubation after plasma treatment (c, d). Statistical comparison was done using one-way ANOVA with *Dunnett* corrections for multiple comparison to untreated control, normalized control (^∗^ *p* < 0.05, ^∗∗^ *p* < 0.01, ^∗∗∗^ *p* < 0.001).

The nuclear localization of p53 is caused by activation of p53 through phosphorylation of serine 15 (Ser15) and serine 37 (Ser37). The phosphorylation levels one hour after plasma treatment showed a clear dependence on treatment intensity. Phosphorylation on Ser15 was increased after 60 s and more clearly after 180 s (Figure 3(a)). In comparison, phosphorylation level of p53 on Ser37 was only slightly increased after 60 s but raised fourfold after 180 s of treatment (Figure 3(b)). On a time axis, a rapid increase in Ser15 phosphorylation, reaching a maximum eightfold increase after 45 min, was observed. This level remained until 6 h after treatment and returned to the baseline level after 24 h (Figure 3(c)). For Ser37 phosphorylation, a slow and constant increase was observed which lasted for more than 24 h (Figure 3(d)). The positive control H_2_O_2_ (100 *μ*M) showed an intensely increased Ser37 phosphorylation, whereas Ser15 phosphorylation level was not affected (data not shown).

### 3.4. Phosphorylation of p53 Upstream Kinases after Plasma Treatment

**Figure 4:**
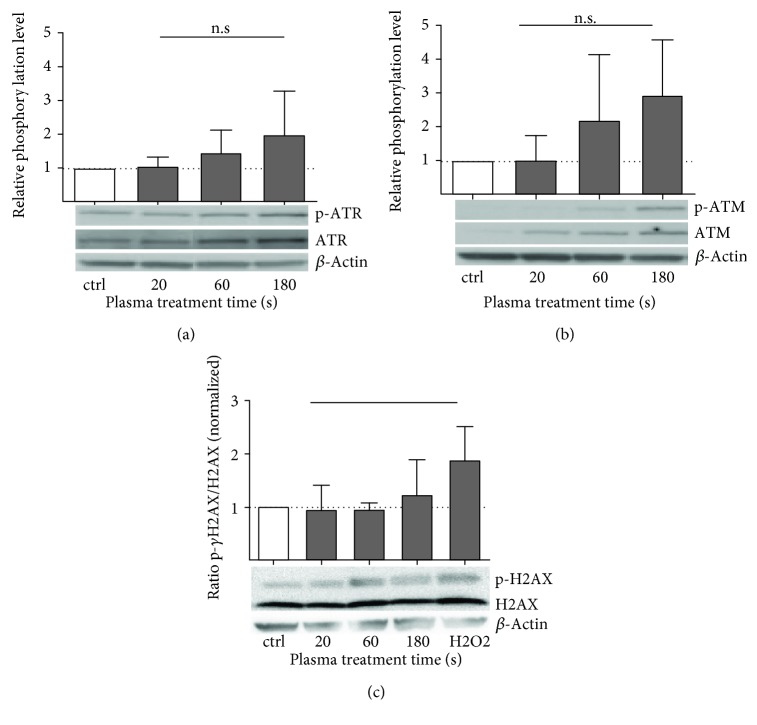
Cold plasma activates upstream activators of p53 regardless of *γ*-H2AX function. Relative phosphorylation of ATR (p-S428) was slightly enhanced in comparison to control (a). Quantification of Western blot has shown a treatment time-dependent induction of phosphorylation of ATM (p-S1981) in HaCaT cells (b, not significant). Phosphorylated H2AX (*γ*H2AX), as a key factor in sensing double strand DNA breaks, was not significantly enhanced after plasma treatment (c). Representative blots are shown. Data are presented as mean + S.D. of two analyses. The *x*-axis represents treatment time with plasma or control (ctrl)/positive control (H2O2). Statistical analysis was done using one-way ANOVA with *Dunnett* corrections for multiple comparisons to untreated, normalized control.

Several sensors and upstream activators of p53 were analyzed regarding their plasma-induced activation pattern. A treatment time-dependent induction of ATR and ATM phosphorylation was observed one hour after plasma treatment (Figures 4(a) and 4(b)). The phosphorylation of ATR/ATM was transient and returned to baseline 24 h after treatment. The control (100 *μ*M H_2_O_2_) led to a very slight rise in p-ATR, whereas the p-ATM levels raised profoundly (data not shown). No impact on phosphorylation status and total protein expression of *γ*-H2AX by indirect plasma treatment was detected (Figure 4(c)).

**Figure 5:**
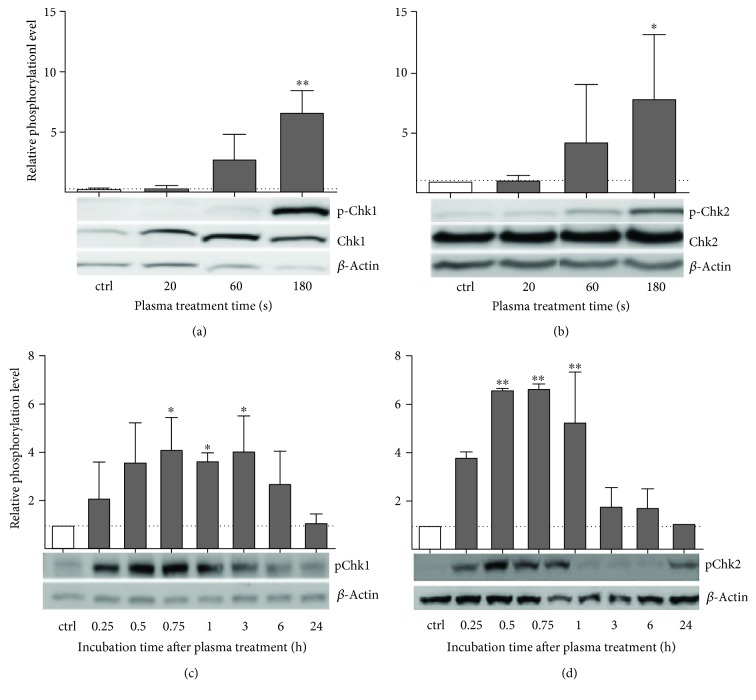
Cold plasma-induced activation of checkpoint kinases 1 and 2 downstream of ATM/ATR in HaCaT cells. Displayed are the increase of the relative phosphorylation levels of Chk1 (a, p-S296) and Chk2 (b, p-T68) after different treatment times, and time course of phosphorylated Chk1 (c) and Chk2 (d) protein after 180 s of plasma treatment over 24 h. Phosphorylated proteins were normalized to *β*-actin. Data are presented as mean + S.D. of three analyses. The *x*-axis represents treatment time (a, b) or incubation after plasma treatment (c, d). Statistical analysis was done using one-way ANOVA with *Dunnett* corrections for multiple comparisons to untreated, normalized control (^∗^ *p* < 0.05, ^∗∗^ *p* < 0.01).

Phosphorylation level of Chk1, the kinase downstream of ATM, was tightly coupled to plasma treatment and incubation time. A more than twofold increase of Chk1 phosphorylation after 60 s and a sixfold upregulation after 180 s of treatment was detected (Figure 5(a)). After treatment, phospho-Chk1 levels increased rapidly and reached a plateau after 45 min (about fourfold increase), where levels remained almost constant for the following hours. Beginning 6 hours after the treatment baseline level was reestablished 24 h postplasma treatment (Figure 5(c)). Chk2, the checkpoint kinase downstream of ATR, was phosphorylated rapidly and transiently. A just over threefold increase after 60 s and more than sevenfold increase in p-Chk2 levels after 180 s of treatment was found (Figure 5(b)). Timewise, the levels of p-Chk2 increased even more rapidly than those of p-Chk1. The maximum was reached around 30 min after plasma treatment (5-fold increase), followed by a rapid decline in p-Chk2 levels so that by three hours after plasma treatment, the levels of p-Chk2 had returned to nearly baseline level (Figure 5(d)). In addition, both molecules were strongly phosphorylated in the H_2_O_2_ control (data not shown).

### 3.5. Cold Plasma Activates Mitogen-Activated Protein (MAP) Kinase Signaling

**Figure 6:**
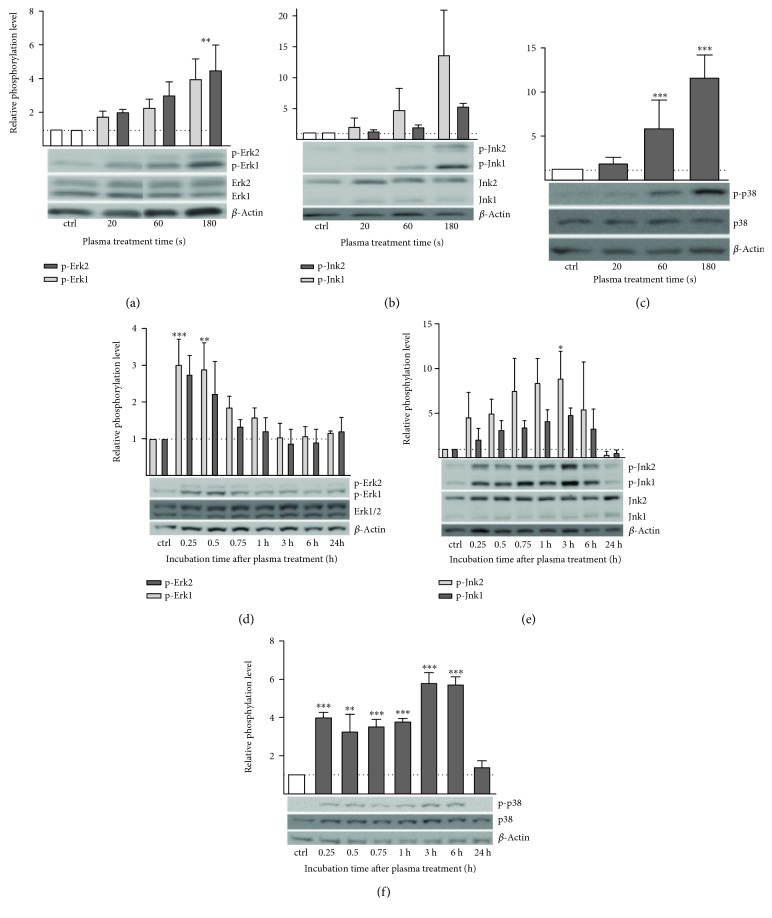
Plasma-induced activation of MAP kinase signaling in HaCaT keratinocytes. Displayed are relative phosphorylation levels of Erk (p-T202/Y204, a), Jnk (p-T183/Y185 (b)), and p38 (p-T180/Y182, (c) kinases after different treatment times. Lower panels showed the results for time course of relative phosphorylation levels after 180 s of plasma treatment for Erk (d), Jnk (e), and p38 (f). Each expression was normalized to total protein expression. Representative blots are shown. Data are presented as mean + S.D. of two analyses. The *x*-axis represents treatment time (a–c) or incubation after plasma treatment (d–f). Statistical analysis was done using one-way ANOVA with *Dunnett* corrections for multiple comparisons to untreated, normalized control (^∗^ *p* < 0.05, ^∗∗^ *p* < 0.01, ^∗∗∗^ *p* < 0.001).

The phosphorylation level of the mitogen-activated protein kinases Erk1/2 (MAPK1/2) increased steadily with treatment intensity and peaked at nearly fivefold increase after 180 s (Figure 6(a)). A similar behavior was observed for p38 and Jnk phosphorylation, which both peak at 180 s of treatment (Figures 6(b) and 6(c)). Unlike the more continuous increase observed for Erk phosphorylation levels, Jnk shows an abrupt escalation in phosphorylation level up to 14-fold after 180 s of treatment compared to 20 s (1.5-fold) or 60 s (4.8-fold). Kinase p38 phosphorylation was increased 7-fold (60 s) and 11-fold (180 s), respectively. The positive control (100 μM H2O2) led to an increase of the phosphorylation levels in all three kinases (data not shown). The time course of the MAP kinase phosphorylation triggered by 180 s of treatment was found to differ between the three kinases; while Erk1/2 phosphorylation increased rapidly, peaking between 15 and 30 min (Figure 6(d)), Jnk phosphorylation levels rose slowly but constantly, peaking after 3–6 h posttreatment. In contrast, p38 levels showed a more flat and biphasic behavior with similar levels up to 1 h past treatment and higher levels for 3–6 h after treatment (Figures 6(e) and 6(f)). The positive control H_2_O_2_ did not lead to increased Erk phosphorylation but induced phosphorylation of p38 and Jnk only (data not shown). All three MAP kinase phosphorylation levels returned to their baseline 24 h after plasma treatment.

### 3.6. Downstream Effects of p53 Activation upon Plasma Treatment

**Figure 7:**
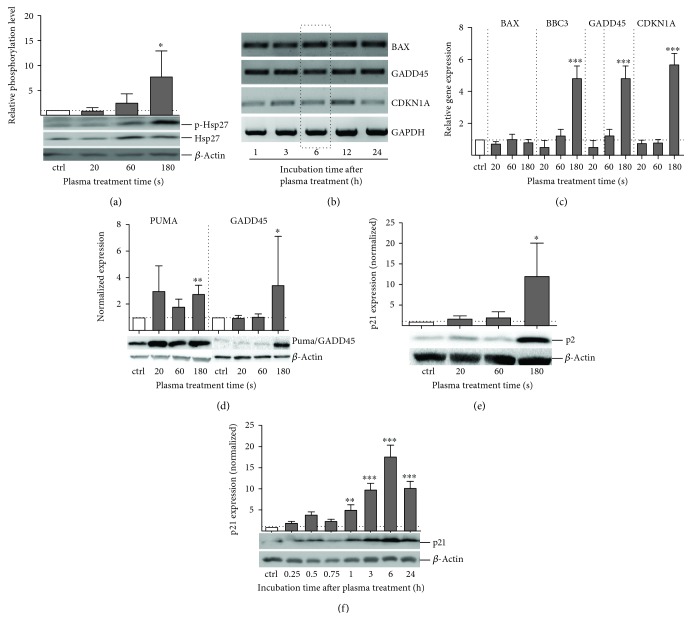
Cold plasma-induced impact on major p53 downstream targets. The total amount and the phosphorylated form of the stress-related protein HSP27 was significantly enhanced after plasma treatment (a). Agarose gel showing semiquantitative PCR of BAX, BBC3, GADD45, and CDKN1A after 1 to 24 h posttreatment of HaCaT cells with 60 s plasma (b). mRNA copy numbers of all four targets were measured 6 h after plasma treatment by qPCR and normalized to the relative gene expression (ΔΔCT values on a log2 scale) (c). Puma (protein of BBC3), Gadd45 (d), and p21 (e) expression was significantly enhanced after plasma exposure (180 s). p21 (protein of CDKN1A) increased until 6 hours after plasma treatment, declining afterwards (f). Representative blots are shown. The *x*-axis represents treatment time (a, c, d, and e) or incubation after plasma treatment (b, f). Data are presented as mean + S.D. of two (c, d) or three (b) analyses. Statistical analysis was done using one-way ANOVA with *Dunnett* corrections for multiple comparisons to untreated, normalized control (^∗^ *p* < 0.05, ^∗∗^ *p* < 0.01, ^∗∗∗^ *p* < 0.001).

To clarify the signaling cascade, we checked the downstream activation of p53 target genes by quantitative real-time PCR (qPCR). The expression levels of BAX and BBC3 (proapoptotic pathway), GADD45 (DNA repair), and CDKN1A/p21 (cell cycle control, senescence) were not significantly altered by 60 s of plasma treatment after 3–12 h (Figure 7(a)). The six-hour time point was chosen for further analysis: a significant, roughly 5-fold increase in BBC3 and GADD45 as well as CDKN1A mRNA expression was induced by long (180 s) treatment. With shorter treatments (20 s and 60 s), target gene expression was slightly but nonsignificantly reduced. BAX expression remained unaltered (Figure 7(b)). Additionally, the cellular protein levels of Bax, Puma (the gene product of BBC3), Gadd45, and p21 (the gene product of CDKN1A) were analyzed by Western blotting. The pattern observed on the transcriptional level was confirmed by an increased expression of BBC3/Puma (Figure 7(c)), Gadd45 (Figure 7(d)), and CDKNA1/p21 (Figure 7(e)) while Bax remained unchanged (data not shown). For p21, a maximal upregulation of protein was visible six hours after plasma exposure (Figure 7(f)). In contrast to transcriptional regulation, Gadd45 protein expression was not altered for investigated time points and treatment times.

**Figure 8:**
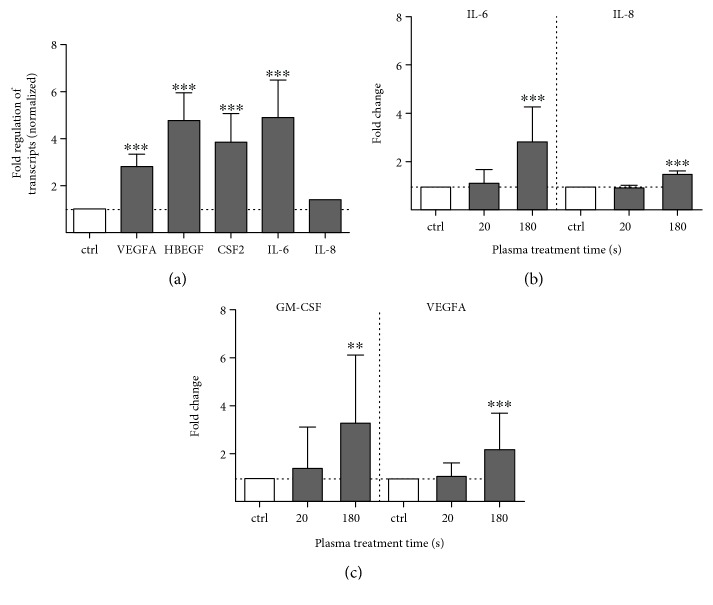
Plasma-induced changes of downstream effectors of MAP kinase signaling cascade in HaCaT keratinocytes. Transcription of several growth factors and cytokines was measured using qPCR. VEGFA, HBEGF, CSF2, and IL-6 were mainly upregulated after plasma treatment in contrast to nonregulated IL-8 (a). Results of secretion of IL-6 and IL-8 (b) cytokines as well as GM-CSF and VEGFA (c) growth factors using ELISA measurements confirmed the mRNA expression profiles. Data in diagrams are presented as mean + S.D. of at least three independent experiments. Statistical analysis was performed using one-way ANOVA with *Dunnett* corrections for multiple testing to untreated, normalized control (^∗^ *p* < 0.05, ^∗∗^ *p* < 0.01, ^∗∗∗^ *p* < 0.001).

The MAP kinase and ROS/RNS-associated heat shock protein 27 (Hsp27), a downstream effector of the p38 signaling cascade, showed a clear correlation of phosphorylation level with treatment time. Similar to the p38, the maximal phosphorylation level was reached after 180 s of treatment (Figure 8(a)). In H_2_O_2_ control, phosphorylation of Hsp27 was only mildly stimulated (data not shown).

Gene expression and protein secretion of several cell-signaling molecules, *e.g.*, growth factors, and cytokines are strongly regulated by the MAP kinase activity. The plasma treatment had a significant impact on mRNA level resulting in a three- to fivefold upregulation of growth factors such as the vascular endothelial VEGFA and heparin-binding growth factor HBEGF as well as the colony-stimulating factor CSF2. For IL-6 as a proinflammatory cytokine, the mRNA levels showed a significant upregulation of DNA transcription, in contrast to IL-8.

The secretion of the two cytokines (IL-6/8) and growth factors (GM-CSF and VEGFA) was further measured using ELISA. Cytokine profiling indicated that IL-6 and IL-8 are most significantly increased in 180 s treated cells after 12 h (Figure 8(b)). The secretion of GM-CSF and VEGFA was found to be maximal 12 h after 180 s of treatment (Figure 8(c))

## 4. Discussion

Cold atmospheric plasma generates a mixture of reactive oxygen or nitrogen species (ROS/RNS) in the gas phase that in contact with liquids lead to the deposition of secondary/tertiary species. Among those, singlet oxygen, atomic oxygen, hydroxyl radicals, and hydrogen peroxide are major contributors [48–50]. Since several years, CAP is used and investigated in biomedical research and clinics, especially for targeting chronic and acute wound management [2, 5, 10, 13, 51]. While it is a long-standing axiom that CAP has antimicrobial properties [52–54], its role and biochemical interaction with mammalian cells are less clear. Depending on treatment intensity and model system, CAP induces cell death and senescence or sparks cell activation and differentiation [16, 55–57]. Latest research seeks to connect CAP with inflammatory processes and immune system control [58–61].

After CAP treatment, an increase of intracellular ROS was observed in HaCaT cells using an unspecific redox-sensitive dye which is in line with previous findings [62, 63]. However, it remains to be elucidated, which reactive species of which origin contribute to the oxidation of intracellular CM-H_2_-DCF (after ester cleavage) since no reporter dyes that are accepted of being able to distinguish between different intracellular ROS are commercially available. Intracellular compartmentalization increases complexity that is not addressed by the simple dye. A promising but demanding approach in this regard is thiol switch dyes (HyPER) [64]. Subsequently, a rapid yet transient increase of total p53 expression accompanied by its nuclear accumulation was observed. Parallel to the nuclear trafficking, serine phosphorylation (Ser15 and Ser37) indicated an activation of p53 via external stimuli, which has been described for UV light stimulation previously [65]. Reports also demonstrate that p53 serine 15/37 sites are phosphorylated by stress-related c-Jun N-terminal kinase (Jnk) and mitogen-activated protein kinase p38 (p38) as well as several upstream kinases, especially ataxia telangiectasia mutated (ATM), ataxia telangiectasia and Rad3-related (ATR), and checkpoint kinase 1/2 (Chk1/2) [66]. Besides DNA damage transduction, ATM and ATR act as cellular redox sensor signals [67–69]. It was found that the ATM protein kinase activity was directly activated after exposure of cells to H_2_O_2_ without the presence of DNA strand breaks [70]. Observations point to the importance of ATM in oxidative stress response regulation in addition to its DNA damage sensing [71]. In an ATM-deficient mice model, increased levels of ROS and signs of oxidative stress within the central nervous system were detected [72, 73]. Predominantly after long CAP treatment, the phosphorylation of ATM and the subsequent activation of the checkpoint kinase Chk1 were observed, which is in good agreement with the finding that ATM is a relevant sensor for reactive oxygen (or nitrogen) species. The direct activation of ATM may trigger protective downstream effects in mammalian cells that potentially cross-correlate with the NFE2L2 pathway [16]. It remains to be established whether ATM signaling occurs upstream of NFE2L2 thiol oxidation and is a necessary precondition or if both pathways are activated in parallel. The fact that the phosphorylation of the histone *γ*-H2AX was only nonsignificantly increased by CAP seems to contribute to the above conclusion as only a strong *γ*-H2AX activation points at DNA damage as initial effect. Additionally, DNA damage was not reported a major route in vitro and in vivo for the plasma device investigated here [74–76]. However, a robust activation of checkpoint kinases 1 and 2 was detected together with an increase of G2 phase cells in cell cycle. This is only in part in agreement to findings reported from a nitrogen plasma jet, which led to a strong *γ*-H2AX phosphorylation in human colon cells, combined with an increase of Chk2 activation [77]. In contrast to the noble gas plasma used in this work, nitrogen plasmas in contact with physiological liquids produce high amounts of peroxynitrite and hypochlorite, facilitating oxidative processes in biomolecules that are subsequently reflected by the activation of *γ*-H2AX seen by the authors. Paralleling the low increase of *γ*-H2AX, only small changes in ATR phosphorylation were seen for all plasma treatment times. This indicates that only a limited and transient oxidative damage to cellular DNA occurred. The activation of ATR is also reflected by the Ser37 phosphorylation of p53 that the kinase can be responsible for; yet, also ATM is able to phosphorylate p53 at Ser37 to a minor extent [69]. On these grounds, it can be argued that plasma treatment triggers redox signaling and such increases the “awareness” of prooxidant species [78]. Indicative for transcriptionally active p53 is the detected transcription of apoptosis pathway proteins (BBC3 and PUMA but not BAX), cell cycle arrest (CDKN1A gene and p21 protein), and DNA repair (GADD45). Of note, previous studies using the kINPen and another noble gas plasma source demonstrated the absence of mutagenic effects in plasma-treated cells [74, 75, 79].

**Figure 9:**
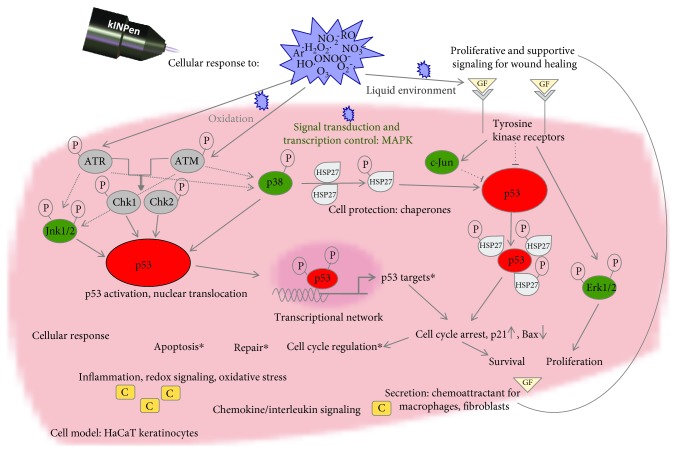
Schema of proposed cold plasma-induced regulation of p53. The primary event in the described pathways is the recognition of plasma-generated reactive oxygen species (ROS) by specific ROS sensors in keratinocytes (*e.g.*, transcription factors p53 and Nrf2 and kinases ATM or Keap1). Plasma generates ROS which in turn activate and phosphorylate p53 via upstream kinases. Activation of p53 increases transcription of p53 targets (BAX, CDKN1A, and GADD45), which increases p53-dependent apoptosis and cell death. Increased expression and phosphorylation of heat shock protein HSP27 by p38 MAP kinase result in p53 binding. HSP27 protects HaCaT cells from plasma-induced apoptosis by increased transcription of p21 resulting in cell cycle arrest, DNA repair, and cell survival. Plasma-induced activation and phosphorylation of MAP kinases (*e.g.*, signal transduction and transcription control) modulates the expression of genes and proteins related to proliferation and cell survival via Erk1/2. Therefore, p53 acts as an anti- and prooxidant.

A connected pathway is the mitogen-activated protein (MAP) kinase signaling. MAP kinases have a direct role in translating physical stimuli into biochemical signals. The phosphorylation of p53 at serine 15 by p38 kinase was detected after UVB irradiation [66]. In addition, CAP treatment as a physical-chemical stimulus was shown to lead to the activation of MAP kinases both in normal and in cancer cells [80–84]. In this work, a time and treatment intensity-related activation profile of the kinases p38, Jnk, and Erk was observed. The activity pattern of the stress-activated protein kinases p38 and Jnk was similar to each other while Erk phosphorylation showed an inverted time course. Notably, Erk activation occurs faster and at lower treatment intensity than Jnk and p38 activation but is less persistent. It can be assumed that this reflects the activity of short-lived reactive species (O, O_2_^−^, ^1^O_2_) at the cell membrane via oxidation of the epidermal growth factor receptor [85] or lipid peroxidation products like oxidized phospholipids [86, 87]. The prolonged activation of Jnk and p38 can be related to H_2_O_2_ which is able to cross the cell membrane via aquaporins [88]. Downstream of the MAP kinase signaling an activation of the antiapoptotic heat shock protein Hsp27 was observed. The molecule serves various functions and acts as a mediator between ROS, p53, and MAPK signaling [89–91]. Hsp27 is also involved in cytoskeleton remodeling and cell migration, which is modulated by CAP treatment [5, 92]. Further, an increase of transcription and secretion of signal molecules involved in wound healing processes and cell migration has been observed. The acute-phase cytokines IL-6 and IL-8 are closely interwoven with MAPK-related signaling [93]. Having chemotactic impact, they also stimulate neutrophil trafficking and T-cell differentiation. IL-6 is the more robustly increased of the two, although also IL-8 is reported to be controlled by ATM [94]. Plasma treatment induces upregulation of GM-CSF and VEGFA in normal keratinocytes, which this study confirmed [81]. An overview of the impact of CAP treatment on the cellular redox signaling based on the present data is given in Figure 9.

There is mounting evidence that p53 modulates the wound healing processes [27], and the data presented here further motivate the use of CAP in wound management. In acute wounds, a transient inhibition of the p53 occurs to support cell proliferation. This could not be observed in the cell model used, instead the activation of the p53 signaling cascade led to a decrease of cellularity that is relevant for wound maturation in order to avoid the development of scar tissue [95, 96]. Along the same avenue, a decrease of cell proliferation and metabolism is welcomed to reduce fibrosis in healing wounds [97]. As discussed, p53 signals in cell migration. CAP resulted in a transiently enhanced migratory activity of normal cells [5, 11]. Finally, the secretion of chemotactic interleukins triggers immune cell invasion and activation. Taken together, CAP treatment leads to a number of functional consequences that are in part due to the concerted action of MAPK pathway and p53 signaling, which was presumably triggered by short-lived reactive species expelled from the plasma source.

## 5. Conclusion

Cold plasma treatment leads to complex, interwoven redox signaling events in mammalian cells via the impact of short- and long-lived ROS/RNS. Signaling related to the p53 axis was found to be a major hub of cold plasma-cell interaction, along with the upstream redox sensors ATM and ATR. Further, MAP kinase signaling accompanied and modulated the p53 signals. Both, negative (p53 activation) as well as positive (Erk activation) effects on cellular longevity were detected for human keratinocytes. A treatment intensity dependence of the observed proapoptotic, proinflammatory, and prosurvival effects as well as a general activation of redox-related proteins showed a timeline dependency of all changes. Functional consequences with regard to the clinical use of plasmas in wound treatment are a reduction in cell proliferation, a transient increase in cell migration, and secretion of immunomodulatory signal proteins.

## Data Availability

No -omics data were used; most other data can be found in the manuscript. Further data (Western blot scans, etc.) are available upon request.

## Abbreviations

ATM: Ataxia telangiectasia mutated
ATR: Ataxia telangiectasia and Rad3-related
Chk1/2: Checkpoint kinase 1/2
Erk: Extracellular signal-regulated kinase
Hsp27: Heat shock protein 27
Jnk: c-Jun N-terminal kinase
MAPK (p38): Mitogen-activated protein kinase
p53: Tumor suppressor protein
RNS: Reactive nitrogen species
ROS: Reactive oxygen species.

## Supplementary Materials

Supplementary Materials Table 1: primer sequences for downstream targets of p53, which were used in quantitative RT-PCR analyses.
