# Supplementary material for: Cold Physical Plasma Modulates p53 and Mitogen-Activated Protein Kinase Signaling in Keratinocytes
Source: Oxid Med Cell Longev. 2019 Jan 13;2019:7017363. doi: 10.1155/2019/7017363 (PMC6348845; doi:10.1155/2019/7017363)
Supplement: Supplementary Materials — Table 1: primer sequences for downstream targets of p53, which were used in quantitative RT-PCR analyses. [file 7017363.f1.pdf]

Suppl. Tables

Table 1: Primer sequences for down-stream targets of p53, which were used in quantitative RT-PCR analyses.

gene	sense primer	antisense primer
**BAX**	TGCTTCAGGGTTTCATCCAG	GGCGGCAATCATCCTCTG
**BBC3**	GACGACCTCAACGCACAGTA	AGGAGTCCCATGATGAGATTGT
**GADD45**	CGCCTGTGAGTGAGTGC	CTTATCCATCCTTTCGGTCTT
**CDKN1A**	CCTCAAAGGCCCGCTCTACATC	GCCCAGCACTCTTAGGAACCTC
**GAPDH**	ACCACAGTCCATGCCATCAC	GGGCTCTCCAGAACATCA
